# Development of a high-throughput growth assay for bacteria or yeasts using an agar-based insoluble carbon source immobilization method

**DOI:** 10.1128/spectrum.04171-25

**Published:** 2026-04-30

**Authors:** Jiabao Liang, Isabella S. Naimi, Achala Narayanan, François Maillard, Peter G. Kennedy, Jeffrey G. Gardner

**Affiliations:** 1 Department of Biological Sciences, University of Maryland - Baltimore County 123999, Baltimore, Maryland, USA; 2 Department of Plant and Microbial Biology, University of Minnesota 172728 , St. Paul, Minnesota, USA; 3 Department of Biology, Lund University 193193, Lund, Sweden; Forschungszentrum Jülich GmbH, Juelich, Germany

**Keywords:** 3D printing, carbohydrate, *Cellvibrio japonicus*, chitin, glycan, pectin, polysaccharide

## Abstract

Polysaccharide degradation by microorganisms is essential in driving global nutrient cycling, developing renewable fuels or chemicals, and promoting human gut health. However, complex polysaccharides are often insoluble, making it challenging to study degradation with soluble enzymes or to measure microbial growth. Several protocols exist to address this challenge, including 3D-printed biomass containment devices or agar capture systems to facilitate the study of insoluble carbohydrate degradation. While these methods are functional, they are constrained by variable substrate loading and time-consuming preparation. To address these shortcomings, a 3D-printed pipette was designed and tested as part of a newly developed agar immobilization method to quantitatively monitor the degradation of insoluble polysaccharides for a 96-well microtiter assay. The pipette and method were validated via two mechanisms. First, growth analyses of *Cellvibrio japonicus* wild-type or *bgl2A*, *cbp2D*, and *cbp2E* single deletion mutant strains were conducted using glucose, barley β-glucan, starch, cellulose, chitin, pectin, galactan, or intact yeast biomass as a sole carbon source to benchmark the new method against previously described methods. Second, the agar pipette was used to screen the growth of bacteria or yeasts capable of utilizing insoluble polysaccharides as the sole carbon source. The 3D-printed pipette and agar immobilization method enabled a faster, more consistent, and cost-effective way for high-throughput screening of bacterial or yeast growth using insoluble carbohydrates, suggesting that it can be a useful tool for environmental and applied microbial research.

**IMPORTANCE:**

The insolubility of many polysaccharides contributes to the challenge of studying how these substrates are deconstructed by diverse microorganisms, which limits our understanding of environmental processes dependent on microbes and slows progress in the development of biotechnologies based on insoluble polysaccharide degradation (i.e., renewable fuels and chemicals). Previous methods to circumvent this challenge are typically time-consuming, resource-intensive, and not compatible with all types of insoluble polysaccharides. Therefore, we designed a 3D-printed pipette as part of an agar immobilization method to enable 96-well microplate microbial growth and enzyme assays using insoluble substrates. Validation of this microtiter assay indicated reduced preparation time and material required for high-throughput screening compared to currently used protocols. Furthermore, the method identified new growth phenotypes for a model saprophytic bacterium and accurately tracked growth patterns from a collection of environmentally derived bacteria and yeasts. These results demonstrate the versatility of the 3D pipette and associated method.

## INTRODUCTION

Environmental microorganisms, including both bacteria and fungi, are the primary drivers of insoluble polysaccharide degradation in nature, and the deconstruction of cellulose, chitin, and other glycans is a critical factor determining microbial niche partitioning (1–3). Furthermore, bioconversion of diverse lignocellulosic and chitinous materials is increasingly becoming a feedstock for industrial applications, such as renewable fuels and chemicals (4–7). Consequently, there has been increasing interest in isolating and characterizing environmental microbes proficient at complex polysaccharide degradation (8–10). However, the insolubility of such substrates presents two major challenges. First, many screening methods use soluble or chromogenic polysaccharide derivatives that do not accurately reflect the chemical and structural properties of true environmental polysaccharides and consequently require substantial secondary screening to identify true degraders (11). While a more recently developed plate-based method uses authentic and untreated lignocellulose as the substrate, it is labor-intensive and technically challenging to implement, resulting in a low-throughput screen (12). The second major challenge relates to measuring microbial growth in liquid culture using insoluble substrates. These substrates can scatter the light source equally or more than the cells suspended in the culture, making optical density measurements inaccurate. Furthermore, alternative methods to measure bacterial growth, such as colony-forming unit counting or protein measurements, are problematic because they require destructive sampling, are not amenable to high-throughput experiments, and data are not collected in real time (13, 14).

To address these major challenges, a new approach using 3D-printed biomass containment devices (BCDs) was developed to enable real-time optical density measurements using complex insoluble polysaccharides (15). These devices are reusable porous capsules that encapsulate insoluble materials in test tubes or Erlenmeyer flasks and sequester the substrate from the light source, leaving it accessible to the growing microbes. The subsequent iteration of the device, a microplate biomass containment device (mBCD), was designed as a porous insert that fits inside the well of a microtiter assay plate (16). Similar to a test tube or flask BCD, insoluble substrates are trapped in the space between the mBCD and the microplate well, keeping the light path clear of obstructions during OD measurement. While this iteration of biomass containment allowed for higher-throughput screening, the mBCD was limited by its pore size. Specifically, this method could not test insoluble substrates (e.g*.,* Avicel, starch, or intact yeast cells) that were powdered or smaller than the 1 mm^2^ pores. A third iteration of the BCD system was created using small agar hemispheres to contain these substrates (17). While successful in containing very small insoluble particulate substrates, this method required considerable preparation time, high concentrations of substrate, and presented challenges with inconsistent substrate loading due to non-homogeneous agar mixtures. Consequently, benchmark measurements for maximum and minimum observable optical density (i.e., the dynamic range of the Y-axis on a growth assay graph) were often narrow, making it difficult to observe subtle growth phenotypes.

The most recent iteration of the BCD system eliminated the 3D-printed insert from the microtiter assay well and replaced it with an agar plug that could be transferred to liquid growth media (18). In this report, we refine this method to enable high-throughput screening in a 96-well microplate format using insoluble substrates with a fine particle size. We have termed this method the Agar Pipette Extraction (APE) assay because the innovation is a custom 3D-printed Agar Pipette (AP) that produces a hole in the agar compatible with microtiter assay plate readers. The AP is reusable, sterilizable, and designed for clean cutting and extraction of an agar plug with high precision from a microtiter well. We validated the APE assay by directly comparing it to the mBCD and agar capture systems (ACS) previously described and observed that the APE assay performed as well or better, specifically in terms of providing an improved dynamic range of optical density measurements. Furthermore, the APE assay is less time-consuming and resource-intensive than previous biomass containment methods. We next performed two proof-of-concept experiments to demonstrate applications of the APE assay. First, we tested wild-type and mutant strains of the saprophytic bacterium *Cellvibrio japonicus* for growth using a wide range of insoluble substrates. Using the APE assay, we observed a growth defect for a *bgl2A* mutant when galactan was the sole carbon source. Second, we screened environmental bacterial and yeast isolates for growth using insoluble substrates and found more variable growth on polymer substrates than on simple sugars. These experiments provide evidence that the APE assay is a substantial improvement over other biomass containment methods and enables rapid, high-throughput growth analyses that may be useful for microbial ecology or biotechnology researchers.

## MATERIALS AND METHODS

### Design and fabrication of 3D-printed Agar pipette

APs were designed using default settings in the programs OpenSCAD 2021.01 (openscad.org) and PreForm 3.42.0.443 (formlabs.com/tools/preform). APs were designed as stacked cylinders (total height: 40 mm; outer TOP diameter: 7 mm; inner TOP diameter: 4 mm; outer BOTTOM diameter: 4.2 mm; inner BOTTOM diameter: 4 mm) with an internal catch pocket and four external guide fins. The internal catch pocket had an X-shaped grid (width: 5 mm; depth: 0.5 mm; height: 0.5 mm) located at the center of the AP to prevent agar plugs from being suctioned into the rubber bulb (Fisher Scientific, cat. no. HS20629AF) and blocking air flow. Four external guide fins (width: 1.1 mm; depth: 0.5 mm; height: 7 mm) assisted in centering the device in the microtiter well during agar plug extraction. APs extract a 4 mm cylindrical agar plug from agar-filled wells of a standard 96-well plate (Corning, cat. no. 3370; well depth: 10.67 mm; well diameter: 6.35 mm;) to enable high-throughput screening of bacterial growth. The stereolithography file for the AP is provided as File S1.

**Fig 1:**
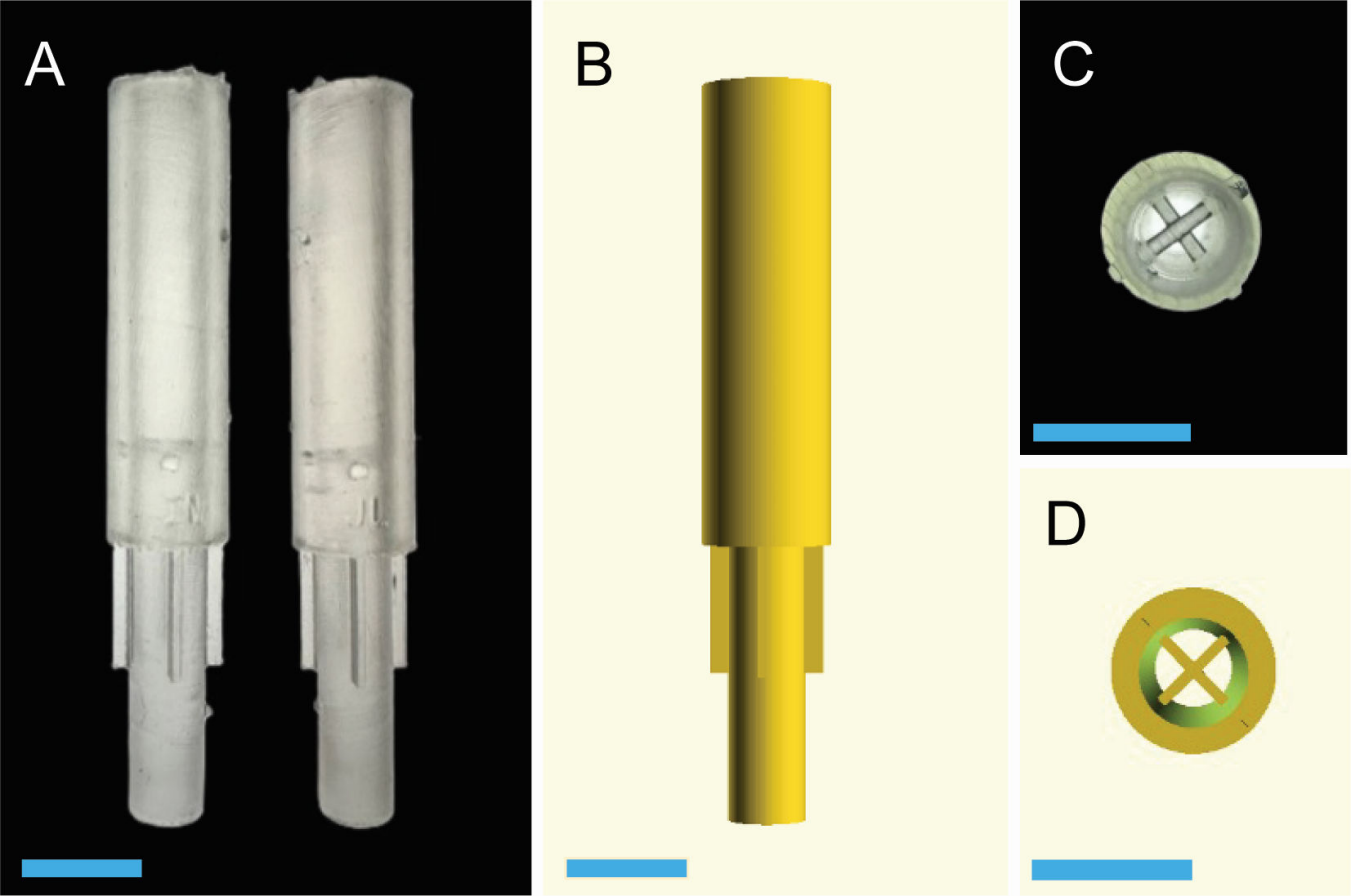
Schematics and 3D prints of the agar pipette. (**A**) Frontal view photograph, (**B**) frontal view schematic, (**C**) bottom view photograph, and (**D**) bottom view schematic. In panels (**A–D**), the blue bar at the bottom of each image corresponds to 7 mm.

Fabrication of the APs used a Form 3+ low-force stereolithographic 3D printer (Formlabs, USA) with clear resin V4 (Formlabs, cat. no. RS-F2-GPCL-04) as per the manufacturer’s instructions. A schematic of the 3D-printed agar pipette and examples of the device are shown in Fig. 1. Ten devices were printed at a time, each with a full support matrix, 12 touchpoints, a 0.1 mm layer depth, and 468 print layers. The APs were cured via UV-irradiation (Formlabs, Form Cure) at 405 nm for 15 minutes at 60°C. Once polymerized, the APs underwent autoclave sterilization using a 30-minute steam cycle at 121°C and 16 PSI without any adverse effects. APs can be reused and re-autoclaved before being discarded due to damage.

### Propagation of bacterial and yeast strains

Wild-type *Cellvibrio japonicus* strain Ueda107 was acquired from the National Collections of Industrial, Marine and Food Bacteria (NCIMB #10462). All *C. japonicus* in-frame deletion mutants were generated through triparental mating as previously described (19). Table S1A lists the genotypes of *C. japonicus* strains used in this study. For propagation, *C. japonicus* strains were grown on 5 mL MOPs minimal media (TeKNovar, cat. no. M2101) supplemented with 0.2% (wt:vol) glucose (TekNova, cat. no. G0520) as the sole carbon source and incubated at 30℃ with high aeration (200 RPM) as done previously (20) in 18 mm sterile glass test tubes. Environmental bacteria and yeast strains (Table S1B) were isolated from *Hyaloscypha bicolor* necromass decomposed for 1 month in a pine-dominated forest stand at Cedar Creek Ecosystem Science Reserve (see reference 21 for details on isolation methods). Strains were grown on 10% tryptic soy agar (bacteria) and potato dextrose agar (yeast) prior to inoculation in experiments.

### Agar-immobilized substrate preparation and growth conditions

**Fig 2:**
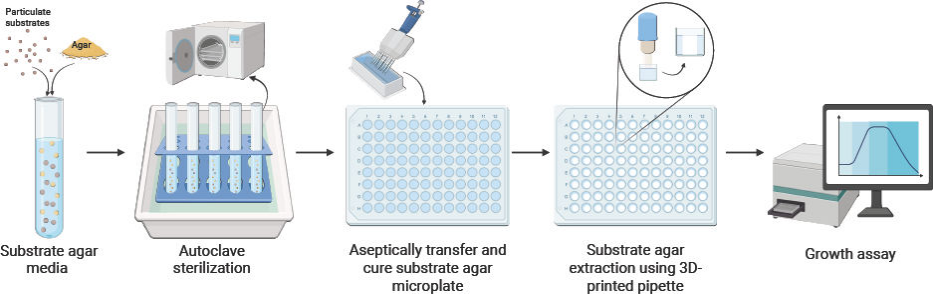
Diagram of media preparation for APE assay. Insoluble particulate substrates are mixed with agar and water, then autoclaved and sterilized in a bin containing water to keep the agar molten. Sterile media components are added post-sterilization to generate a complete minimal medium that is aseptically transferred to a 96-well plate. The agar is allowed to cool and solidify, after which an agar pipette removes an agar plug, which is discarded. A sterile carbon source-free medium is added to the well and then is inoculated. The figure was generated using assets from BioRender. Liang, J. (2025) https://BioRender.com/77k779a.

Substrate-containing agar microplates were prepared similarly to the agar capture system previously described (17) with the following modifications. As depicted in Fig. 2, a substrate medium at four times the final desired concentration was mixed with 3% (wt:vol) agar (Fisher Scientific, cat. no. BP1423) in a test tube and autoclave sterilized (121°C for 30 minutes; liquid cycle). To slow down agar solidification post-autoclave, the tubes were submerged in a bin filled with water that was also autoclaved. For *C. japonicus* experiments, glucose was used as a soluble control carbon source at a final concentration of 0.2% (wt:vol), and complex substrates were used at the following final concentrations: 0.2% (wt:vol) barley β-glucan (MegaZyme, cat. no. P-BGBM); 0.2% (wt:vol) potato starch (Fisher Scientific, cat. no. 50-136-8532); 2% (wt:vol) autoclaved *Saccharomyces cerevisiae* intact cells (ATCC# 204508); 0.2% (wt:vol) pectin, from apple (Sigma, cat. no. 76282-100G); and 0.2% (wt:vol) galactan from potato (MegaZyme, cat. no. P-GALPOT). For substrates with a pH of 5.0 or lower, the substrate and agar media were sterilized separately and mixed after autoclaving. The substrate pH was measured using full-range pH paper (Hydrion; cat. no. JH-613). The substrate-agar mixture was transferred to a sterile reagent reservoir and aseptically dispensed into a sterile 96-well flat-bottom microplate (Corning, cat. no. 3370) in 135 µL aliquots using a multichannel pipette. The microplate was covered and left to rest at room temperature (25°C) for 2 hours. Then, the seam between the lid and plate was sealed with parafilm (Amcor; cat. no. PM-999) and stored at 4°C overnight (~12 hours) before being used for growth experiments. Once fully set, the substrate-containing agar plate can be stored at 4°C. Plates stored up to 3 days at 4°C were used for experiments; however, longer storage times are possible if the plates are covered with a lid, the seams between lid and plate sealed with parafilm, and stored inverted.

To prepare the substrate-agar microplate for growth experiments, a sterile 3D-printed AP attached to a rubber bulb (Fisher Scientific, cat. no. HS20629AF) was used to extract a cylindrical agar plug from the center of the well, leaving approximately 50 µL of substrate agar. Individual wells were then supplemented with 148 µL MOPs minimal media (TeKNova, cat. no. M2101) without any carbon source, followed by inoculation at a 1:100 dilution from an overnight bacterial culture. Control growth experiments using the agar capture system were performed as previously described (17). Bacterial growth was measured via absorbance readings at 600 nm (OD_600_) using a BioTek EPOCH 2 plate reader set at 30°C with high aeration (205 RPM). To prevent evaporation during experiments, two major steps were taken. First, the outermost wells of the plate (i.e., columns 1 and 12; rows A and H) were filled with 200 µL sterile water and not used for experimental measurements. Second, the plates were incubated with a lid and the seam sealed with parafilm. For longer-term experiments, a humidified growth chamber (>50% humidity) also helps prevent evaporation from the plate. All AP growth experiments were performed in biological triplicate. Calculation of growth rate and standard deviation was performed using GraphPad Prism 9 software with default settings.

For experiments on environmental microbes, the same protocol was used with minor modifications. Glucose was also used as a control carbon source, mixed with 3% (wt:vol) agar at a concentration of 0.2% (wt:vol). Other test substrates, also combined with 3% (wt:vol) agar at a concentration of 0.2% (wt:vol), included chitin from shrimp cells (Sigma Aldrich C7170), β-1,3-glucan from *Euglena gracilis* (Sigma Aldrich 89862), and necromass prepared from freeze-dried *Agaricus bisporus* stipes. Substrates were dispensed into microplates, and the AP was used as described above. Microplates were then filled with 140 µL of M9 basal media (1× M9 salts solution amended with CaCl_2,_ trace elements solution, FeCl_3,_ and MgSO_4_). Colonies grown on petri plates were suspended in 500 µL of basal media, and 10 µL was inoculated into each well. Microbial growth was measured via absorbance readings at OD_600_ using a SpectraMax i3x plate reader (Molecular Devices). Measurements were taken daily over the course of a week.

### Biomass contaminant device preparation and growth conditions

Growth experiments using insoluble particulate substrates, including filter paper (1% wt:vol) (BioRad, cat. no. 1703965) and β-chitin extracted from squid pen (1% wt:vol) (France Chitin; Batch 20140101), were performed with biomass contaminant devices (BCDs), following a previously established method (15). Briefly, BCDs containing 50 mg of insoluble substrate were placed in 18 mm glass test tubes and autoclave sterilized (121°C and 16 PSI for 30 minutes; gravity cycle). Then, 5 mL of MOPs minimal media without any carbon source was added to each tube, followed by inoculation at a 1:100 dilution from an overnight bacterial culture. Cells were grown at 30°C with high aeration (200 RPM), and optical density measurements were obtained at 600 nm with a Spectronic 20D+ spectrophotometer (Milton Roy). All BCD growth experiments were performed in biological triplicate. GraphPad Prism 9 software with default settings was used to calculate growth rate and standard deviation.

### *N*-acetylglucosaminidase assay preparation and procedure

The digestion reaction was performed by incubating 1 µL of β-*N*-Acetylglucosaminidase S (New England Biolabs, cat. no. P0744L) in 0.18% (wt:vol) 4-nitrophenyl *N*-acetyl β-D-glucosaminide (Millipore Sigma, cat. no. N9376) mixed with 3% (wt:vol) agar prepared via the APE assay, as described above. Each sample was supplemented with 1× Glycobuffer (5 mM CaCl2, 50 mM sodium acetate). A_405_ values were measured every 15 minutes for 2 hours using a BioTek EPOCH 2 plate reader set at 37°C without shaking. Three negative controls were subjected to the reaction: no enzyme, no substrate, and no enzyme or substrate. All enzymatic assays were performed in biological triplicate. GraphPad Prism 9 software with default settings was used to plot the absorbance curves.

### Cornstarch digestion and glucose detection using GAGO assay

A substrate-agar microplate containing 0.2% (wt:vol) glucose, 0.5% (wt:vol) insoluble cornstarch (Signature SELECT), or no additional carbon (agar only) was prepared as described above. Microplate wells were then supplemented with 148 µL of MOPs minimal medium and incubated at 40°C for 24 hours with or without 2 µL (6 U) of thermostable α-amylase (MegaZyme, cat. no. E-BSTAA). After incubation, 100 µL of samples was collected from each well, and glucose was quantified using a glucose assay kit (Sigma-Aldrich, cat. no. GAGO20-1KT) as per the manufacturer’s instructions. All enzymatic assays were performed in biological triplicate.

## RESULTS

### Validation of APE method

**Fig 3:**
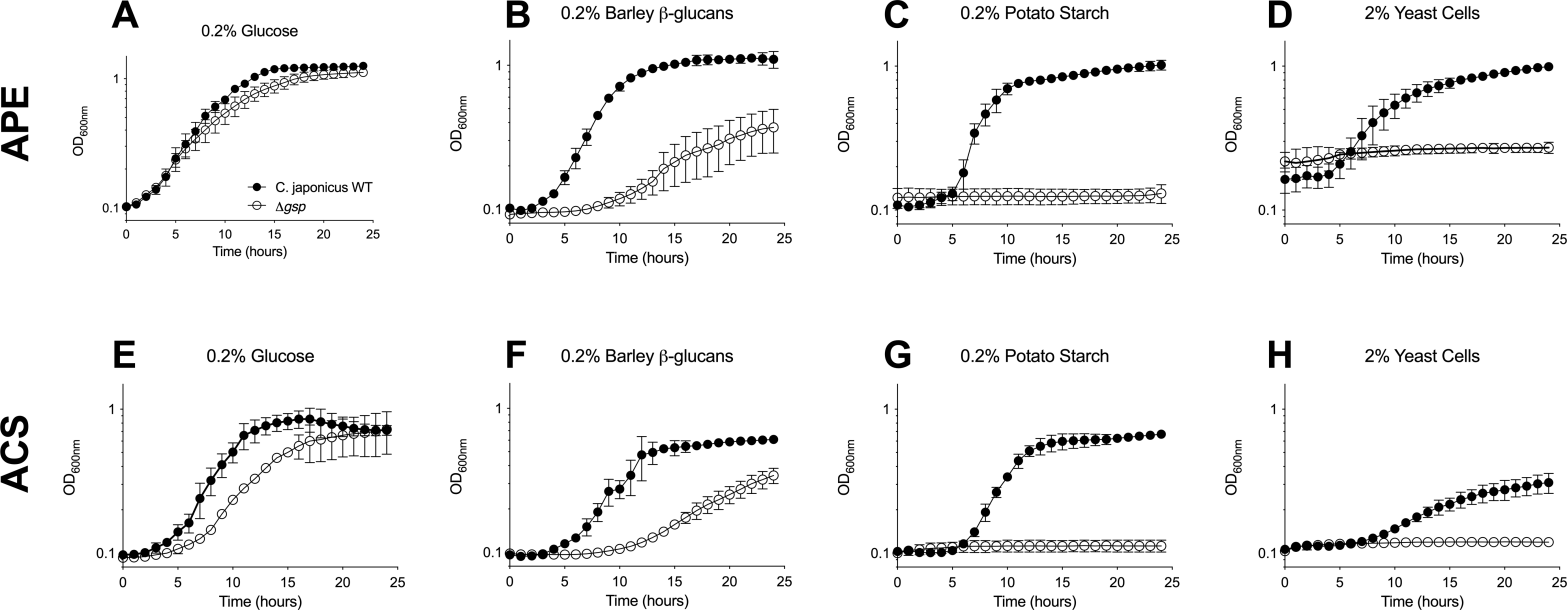
Comparative growth analysis of wild-type *C. japonicus* and *∆gsp* mutant in insoluble substrates using APE versus ACS with mBCDs assay. *C. japonicus* cells were grown in MOPs minimal medium containing either (**A and E**) 0.2% (wt:vol) glucose, (**B and F**) 0.2% (wt:vol) barley β-glucan, (**C and G**) 0.2% (wt:vol) potato starch, or (**D and H**) 2% (wt:vol) autoclaved yeast cells. All growth experiments were performed in biological triplicate with error bars representing standard deviation. Data shown in panels **A, B, E, and F** were collected from independent growth experiments (i.e., different microtiter assay plates). Data shown in panels **C and G** were collected from the same microplate growth experiment. Data in panels **D and H** were collected from the same microplate growth experiment. Panels are shown together for ease of visualization and interpretation.

To benchmark the APE assay in comparison to the agar capture system that uses mBCDs for high-throughput screening of bacterial growth on complex insoluble substrates, we compared the growth of *C. japonicus* wild-type and a *∆gsp* mutant strain using both methods. Insoluble substrates tested included barley β-glucan, potato starch, and autoclaved *Saccharomyces cerevisiae* yeast cells, which resulted in a light brown coloration of the agar. Glucose was used as a soluble positive control substrate. Using the APE assay, we observed that wild-type *C. japonicus* grew well in all four tested substrates, while the *∆gsp* mutant experienced a modest growth defect in barley β-glucan and was unable to grow on potato starch or yeast cells (Fig. 3A through D; Table S2). The growth defects observed for the *∆gsp* strain were expected because previous studies reported that this mutant lacks the Type II Secretion System, which is essential for extracellular Carbohydrate-Active Enzyme (CAZyme) export (19, 20). Furthermore, these phenotypes were previously observed in the study that reported the mBCD method (17). We replicated those experiments again here and found similar growth trends for both strains (Fig. 3E through H; Table S2). Notably, when comparing the two methods, we observed a reduction of the maximum OD_600_ for the wild-type strain, which consequently contributed to a lower upper boundary of measurable growth (i.e., a reduced dynamic range between WT and *∆gsp* observed across all four tested substrates). When yeast cells were used as the sole carbon source, the initial OD_600_ readings in the APE assay are ~1.5× higher compared to the agar capture system, which may be due to the brown coloration of the substrate agar impacting the OD_600_ measurement. However, these benchmark growth experiments demonstrated that the APE assay facilitated quantitative measurement of bacterial growth over a wide range of complex insoluble polysaccharides with a precise and dynamic range that matched or exceeded that of the current methods.

### APE assay growth analyses identify essential CAZyme-Encoding genes required for complex polysaccharide utilization in *C. japonicas*

**Fig 4:**
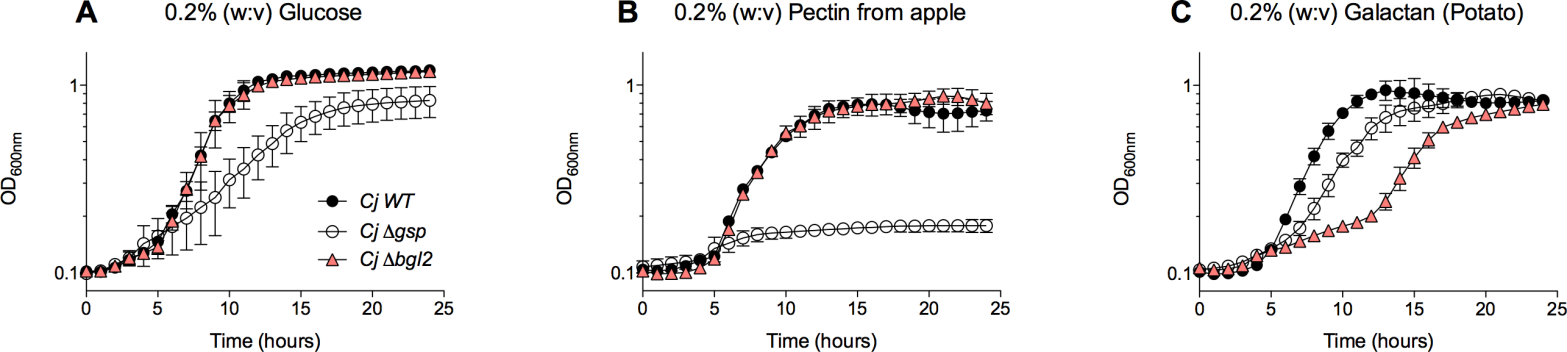
Growth analysis of a *C. japonicus ∆bgl2A* mutant strain using the APE method. Wild-type and a *∆gsp* mutant were shown as positive and negative controls. Cells were grown in MOPs minimal medium containing (**A**) 0.2% (wt:vol) glucose, (**B**) 0.2% (wt:vol) pectin from apple, or (**C**) 0.2% (wt:vol) galactan from potato as the sole carbon source. All growth experiments were performed in biological triplicate with error bars representing standard deviation.

*Cellvibrio japonicus* is well known for its robust ability to completely degrade complex, recalcitrant polysaccharides (22). This bacterium has over 300 CAZyme-encoding genes (including 132 genes that encode polysaccharide-degrading glycoside hydrolases); however, the physiological role of many CAZymes remains unknown (23, 24). Recent studies on *C. japonicus* employed genomic analysis and high-throughput screening of mutant strains to identify and characterize the role of CAZymes during polysaccharide degradation and utilization (25–27). Interestingly, one gene that was significantly up-regulated under several different conditions was the *bgl2A* (CJA_0496) gene. Annotated as encoding a β-galactosidase, the *bgl2A* gene is predicted to play a role in pectin utilization and is adjacent to an *endo*-1,4-galactanase, *gal53A-2* (CJA_0497), and a TonB-dependent receptor (CJA_0498) (28). We generated a ∆*bgl2A* mutant to use in combination with the APE assay as a proof-in-concept to identify growth conditions where the gene was critical. As expected, the ∆*bgl2A* mutant grew like WT in glucose medium (Fig. 4A). While the ∆*bgl2A* mutant strain grew like WT on apple pectin, a reproducible growth defect was observed on potato galactan (Fig. 4B and C; Table S3). *C. japonicus* has two more predicted β-galactosidases (*bgl2B* and *bgl2C*), and ongoing work using the APE assay coupled with pectin and galactan substrates will further characterize how they contribute to hemicellulose deconstruction.

**Fig 5:**
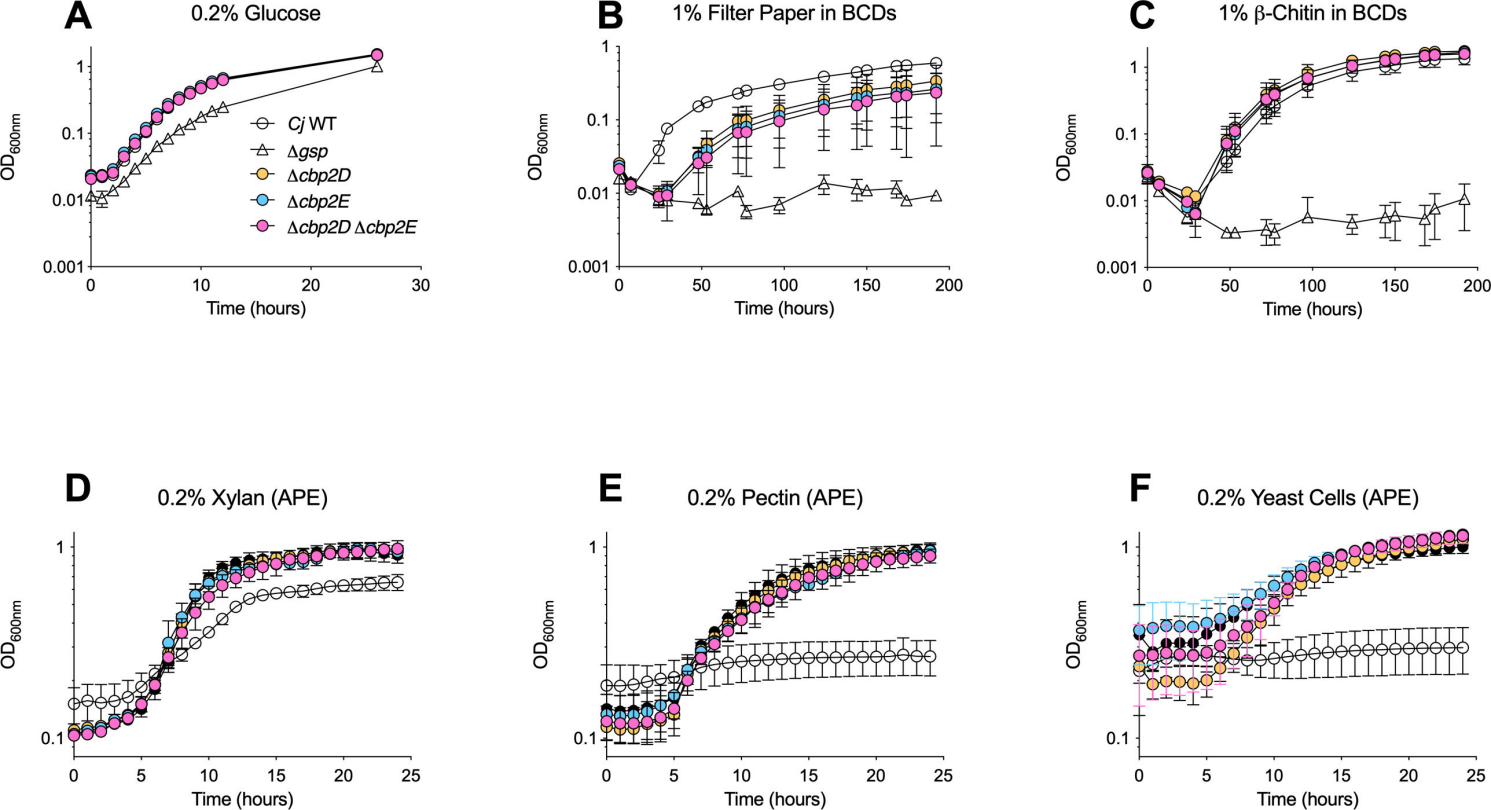
Growth analysis of *C. japonicus* mutant strains in complex carbohydrates using either a microplate with the APE assay (**D–F**) or a test tube with biomass contaminant devices (**A–C**). Cells were grown in MOPs minimal medium containing (**A**) 0.2% (wt:vol) glucose, (**B**) 1% (wt:vol) filter paper, (**C**) 1% (wt:vol) β-chitin, (**D**) 0.2% (wt:vol) xylan, (**E**) 0.2% (wt:vol) pectin, or (**F**) 2% (wt:vol) autoclaved yeast cells as the sole carbon source. All growth experiments were performed in biological triplicate with error bars representing standard deviation. Data presented in panels **A–F** were collected from separate, independent experiments and are shown together for ease of visualization and interpretation.

Two additional genes also reported as significantly up-regulated under multiple conditions in *C. japonicus* RNAseq experiments are *cbp2D* and *cbp2E* (29). These carbohydrate-binding proteins (Cbp2D and Cbp2E) function as electron donors to provide redox power for a lytic polysaccharide monooxygenase to break glycosidic linkages. Additionally, a recent study hypothesized that Cbp2D and Cbp2E work together as a heterodimer to directly cleave linkages during cellulose destruction (30). To test whether the Cbp2D/E proteins both had redox functions important for complex polysaccharide degradation, a ∆*cbp2D* single mutant, ∆*cbp2E* single mutant, and ∆*cbp2D* ∆*cbp2E* double mutant were tested on five complex polysaccharide substrates using the APE method (Fig. 5; Table S4). As expected, all *C. japonicus* mutants grew like wild type in soluble glucose control medium (Fig. 5A). When growing on filter paper (cellulose) using the BCD method, we observed a modest growth defect and reduced final OD_600_ for the *∆cbp2D* and *∆cbp2E* single mutant compared to wild type, as reported in previous studies (Fig. 5B). Interestingly, the deletion of both *cbp2D* and *cbp2E* did not result in a growth defect different from either single mutant. Both single mutants and the double mutant grew like wild type when β-chitin was used as the sole carbon source (Fig. 5C). When grown on a wide range of complex polysaccharides, including xylan, pectin, and autoclaved yeast cells, no growth defect was observed for any Cbp2D/E single or double mutants using the APE assay (Fig. 5D through F). Overall, the mutational growth analyses with *bgl2A* or *cbp2D/E* mutants illustrate the utility of the APE system to rapidly screen a diverse range of polysaccharide substrates to uncover growth phenotypes that deviate from WT.

### APE system for environmental screening of bacterial and yeast isolates enables comparison of growth on simple and complex substrates

**Fig 6:**
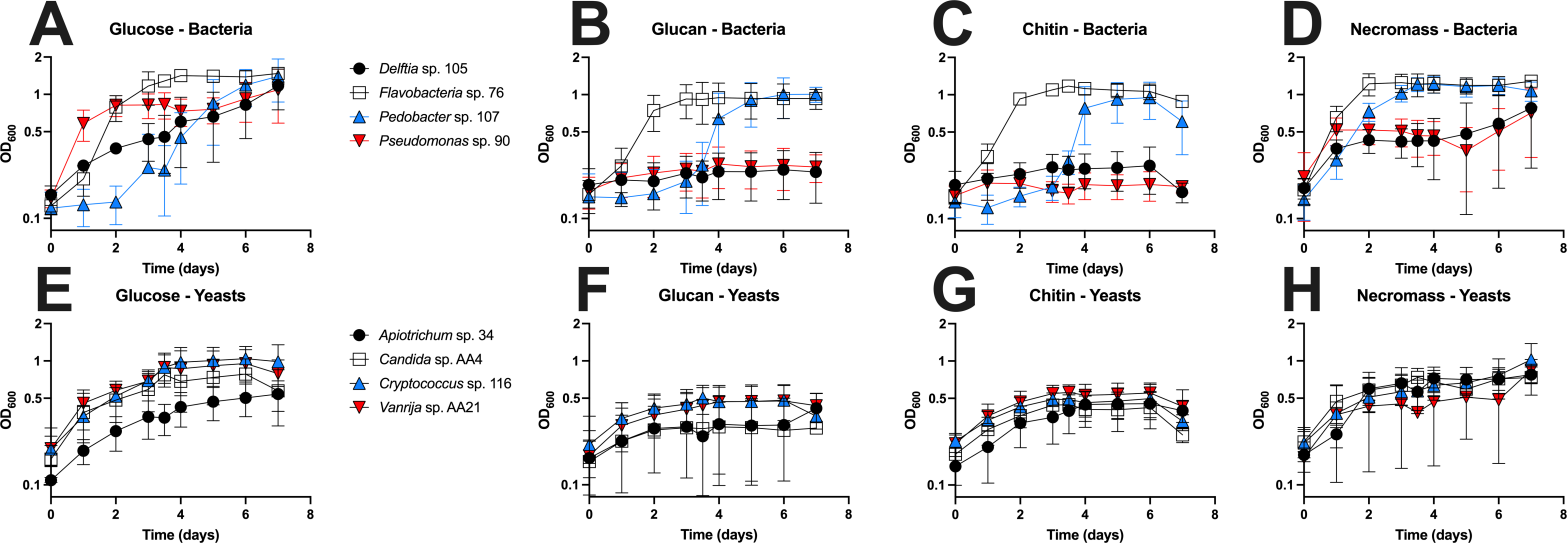
Growth analysis of environmental microbes in complex carbohydrates using microplates with the APE assay. Growth dynamics of bacterial strains (**A–D**) and yeast strains (**E–H**) are shown. Cells were grown in M9 minimal medium containing either 0.2% (wt:vol) chitin, 0.2% (wt:vol) glucan, 0.2% (wt:vol) glucose, or 0.2% *A. bisporus* necromass. All growth experiments were performed with at least six technical replicates and three biological replicates. Error bars represent the standard deviation.

To demonstrate the broad applicability of this assay, we screened the growth of eight environmental isolates (four bacteria and four yeasts) on glucose, chitin, β-glucan, and fungal necromass, which represent a range of carbon sources encountered in soil. As anticipated, all strains exhibited the greatest growth on glucose, with the exception of the fungal *Apiotrichum* strain, which grew most on necromass (Fig. 6). In many cases, the maximum OD_600_ achieved by microbial strains on *A. bisporus* necromass and glucose was relatively similar, though the growth trajectories appeared different. In general, yeasts had lower maximum growth compared to bacteria on insoluble polymers chitin and glucan, with marginally higher growth on chitin. In contrast, both the *Flavobacterium* and *Pedobacter* strains demonstrated a capacity for growth on chitin and glucan, though growth rates were slower and yields were lower than on necromass or glucose. In particular, it appeared that the lag time of the *Pedobacter* strain was prolonged on the polymers relative to the simple sugars. Neither the *Delftia* strain nor the *Pseudomonas* strain displayed any ability to grow on insoluble chitin and glucan.

### APE assay facilitates the high-throughput quantification of β-*N*-acetylglucosaminidase activity

**Fig 7:**
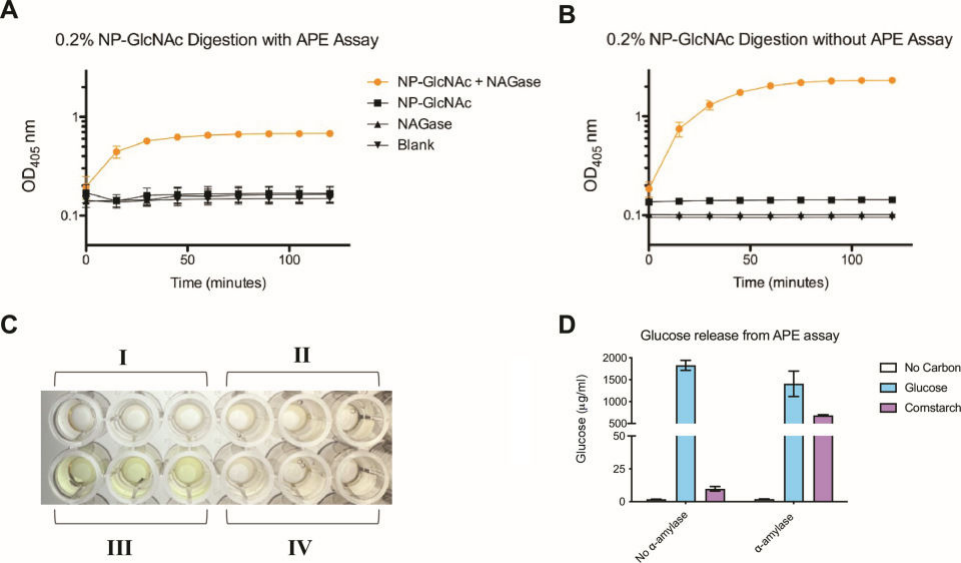
Quantitative measurement of polysaccharide degradation using the APE method. (**A**) Quantitative measurement of 0.2% 4-nitrophenyl *N*-acetyl β-D-glucosaminide (NP-GlcNAc) degradation by β-*N*-acetylglucosaminidase S (NAGase) via the APE assay. The reaction was performed in biological triplicate with error bars representing standard deviation. Enzyme activity is further illustrated in (**B**), containing (i) no NP-GlcNAc or NAGase, (ii) NP-GlcNAc without NAGase, (iii) NP-GlcNAc with NAGase, and (IV) NAGase without NP-GlcNAc. The presence of yellow indicates the production of the cleavage product, 4-nitrophenol. (**C**) Quantitative measurement of 0.2% NP-GlcNAc degradation by NAGase without the APE assay. (**D**) Quantification of degradation products from insoluble cornstarch. Agar containing no additional carbon source, 0.2% (wt:vol) glucose, or 0.5% (wt:vol) insoluble cornstarch was incubated in MOPs minimal media, with or without α-amylase. Degradation products were collected from the center of the well and quantified using a GAGO assay kit.

To determine whether the APE assay supports high-throughput enzymatic assays, we performed a digestion reaction of the chromogenic substrate 4-nitrophenyl *N*-acetyl β-D-glucosaminide (NP-GlcNAc) using β-*N*-Acetylglucosaminidase S (NAGase), an exoglycosidase responsible for the cleavage of terminal *N*-acetylglucosamine residues. Upon cleavage of the internal glycosidic linkage connecting *N*-acetyl β-D-glucosaminide to 4-nitrophenyl by NAGase, the cleavage product 4-nitrophenol is released into the center of the well and can be measured at 405 nm. As shown in Fig. 7B, the addition of NAGase to the experimental wells created a color change from clear to yellow, indicating the successful cleavage of NP-GlcNAc. This result is further supported by the quantification of the cleavage product, 4-nitrophenol (Fig. 7A). To serve as an additional control and estimate the degree to which agar suspension hinders soluble substrate diffusion to the center of the well, the digestion reaction was replicated without the APE assay in the form of liquid NP-GlcNAc media. Figure 7C illustrates that after 2 hours of incubation, the maximum OD_405_ achieved by the addition of NAGase to NP-GlcNAc is approximately three times higher than that of the digestion reaction conducted via the APE assay, indicating that agar diffusion is a limiting factor in such enzymatic assays. To demonstrate that the APE method supports high-throughput enzymatic assays with insoluble substrates, a digestion reaction of agar-trapped insoluble cornstarch with a thermostable α-amylase was performed. As shown in Fig. 7D, glucose was successfully detected from agar-trapped cornstarch treated with α-amylase, but only background levels of glucose were detected in the absence of α-amylase. Thus, in addition to its utility in identifying growth phenotypes, the APE assay can be used to quantify the enzymatic degradation of complex, insoluble polysaccharides in a high-throughput manner.

## DISCUSSION

Due to their industrial, biotechnological, and ecological applications, the study of insoluble polysaccharide degradation by environmental microbes is gaining significant interest (4–7). However, these studies are often hindered by using modified substrates, destructive sampling, low-throughput screening, or substrate light scattering (11–14). To combat these challenges, BCDs and microplate biomass containment devices (mBCDs) were utilized to sequester large insoluble particulates in liquid cultures from the light source, thereby enabling a more accurate and cost-effective method for real-time spectrophotometer measurements (15, 16). To address the large pore sizes within these devices, finer substrates were trapped in agar-hemispheres for high-throughput screening (17). Here, we introduce a refined method for screening of fine, insoluble polysaccharide degradation in a 96-well microplate format known as the APE assay. We assessed the applicability of the novel 3D-printed AP for measuring cell growth and enzyme activity, as well as for screening environmental isolates. To validate the APE assay, we benchmarked it against the growth of *C. japonicus* on a wide range of complex insoluble substrates measured using mBCDs in conjunction with the agar capture system (ACS).

Our proof-of-concept growth experiments demonstrate that the APE assay enables improved, reproducible, high-throughput bacterial growth analysis while eliminating the drawbacks of the ACS. For example, the substrate medium must be prepared at ten times the final desired concentration for the ACS, while only four times higher for the APE assay, making growth experiments more cost-effective. The APE assay is also less time-consuming, as the preparation time is reduced by more than half compared to the ACS. Specifically, the substrate-agar microplate only needs to be prepared 24 hours before the growth experiment, while the ACS with mBCDs requires curing in a high-humidity environment for at least 48 hours before use. In addition to being cost-effective and time-saving, the APE assay provides improved dynamic range, making it easier to detect subtle growth defects. Figure 3 illustrates that the wild-type strain experienced a modest reduction in max OD_600_ measurements when grown on glucose, barley β-glucan, and potato starch in conjunction with the ACS, and a more drastic decrease when yeast was used as the sole carbon source. This contributed to a smaller dynamic range, making it more difficult to differentiate between growth trends in the wild type compared to the ∆*gsp* mutant. While growth defects in ∆*gsp* were expected due to previous experiments, these observations suggest that, compared to the APE assay, the ACS can be less easily utilized to form clear conclusions about unknown CAZyme activity (19, 20). Therefore, the APE assay provides a more versatile and precise method for quantifying bacterial growth on a host of complex insoluble substrates, with a dynamic range that exceeds current protocols.

To demonstrate the application of the APE assay for identifying mutant phenotypes, we measured the growth of *C. japonicus* mutants on several insoluble polysaccharides. Previous RNAseq studies demonstrated that the genes *bgl2A*, *cbp2D*, and *cbp2E* are significantly upregulated during polysaccharide degradation in this bacterium, but the exact function of their enzyme products is unknown (28, 29). Pectin is a galacturonic-rich polysaccharide commonly found in plant cell walls (31). *C. japonicus* has 12 glycoside hydrolase (GH) enzymes predicted to play a role in pectin degradation, including three (*bgl2A*, *bgl2B*, and *bgl2C*) from the GH2 family that encodes a β-galactosidase (28). None of these GH2 enzymes had previously been characterized, and their physiological role in *C. japonicus* pectin degradation remained unclear. Our high-throughput screening of the ∆*bgl2A* mutant on pectin suggests that Bgl2A is not required for pectin degradation (Fig. 4B). This was not surprising given that both Bgl2A and Bgl2B are predicted to be in the periplasm, indicating that these CAZymes may be functionally redundant in *C. japonicus*. The inability of the ∆*gsp* mutant to grow on pectin demonstrates that secreted CAZymes are essential for pectin consumption. A modest growth defect was obtained when the ∆*bgl2A* mutant was grown on potato galactan, illustrating that Bgl2A is important for *C. japonicus* to completely degrade and utilize galactan (Fig. 4C).

The ∆*cbp2D* and ∆*cbp2E* single mutants exhibited the expected growth defect when filter paper functioned as the sole carbon source (Fig. 5B). The ∆*cbp2D*/*2E* double mutant displayed a similar phenotype to the single mutants, supporting a previous study that hypothesized that these two carbohydrate-binding proteins work together as a heterodimer to degrade cellulose (30). Therefore, this study confirms that deleting both proteins is equivalent to deleting either one. Neither of the Cbp2D/2E deficient mutants grew differently from wild-type when grown on non-cellulosic polysaccharides (Fig. 5C through F), suggesting that these proteins bind specifically to cellulose alone.

In addition to its utility in identifying mutant phenotypes through trends in bacterial growth, the APE assay also supports the study of enzymatic activity. By incubating agar-trapped cornstarch in liquid medium with α-amylase, we have demonstrated that the suspension of fine, insoluble polysaccharides via the APE method does not hinder the activity of degradative enzymes (Fig. 7D). Quantitative (Fig. 7A) and qualitative (Fig. 7B) results for the degradation of NP-GlcNAc illustrate a similar result and indicate that even for soluble polysaccharides, agar suspension slows substrate diffusion to the center of the well significantly. By using a chromogenic substrate, we have also demonstrated that the APE assay is both compatible and highly sensitive to the presence of color in the center of the well, as the color change from clear to yellow did not interfere with quantifying the level of NAGase activity. Thus, the APE assay has the potential to be applied to future studies in enzyme kinetics, activity, and expression in mutant strains when incubated with complex polysaccharides.

Expanding the microbes tested to include environmental isolates (Fig. 6), we have also verified the APE assay was functional in capturing the growth dynamics of several bacteria and yeasts. We tested growth on (i) glucose as a control labile sugar source (ii), chitin and glucan, both insoluble polymers prevalent in fungal cell walls, and (iii) necromass derived from *A. bisporus*, consisting of a heterogeneous mixture of cell wall polymers and simple sugars (32, 33). Unsurprisingly, given these strains were isolated from necromass, we found a high degree of growth across strains on both necromass and glucose. Bacteria and yeasts tended to reach the highest maximum growth on glucose, though the *Apiotrichum* strain, a dimorphic yeast with the potential to produce hyphae in liquid culture (34), grew best on necromass. There appeared to be weak growth of yeasts on either glucan or chitin, consistent with the characterization of forest soil yeasts as being primarily opportunists, with limited ability to degrade complex carbohydrates (35). While two of the four bacteria surveyed grew little on either chitin or glucan, both the *Flavobacterium* and *Pedobacter* species exhibited substantial growth on these substrates. A number of *Flavobacteria* species are known to use complex carbohydrates (36, 37), including *Flavobacterium johnsoniae* strains, which can digest insoluble chitin (38, 39). *Pedobacter* species isolated from soil also possess a suite of carbohydrate-active enzymes (40), though prior experiments have found that this genomic potential does not necessarily correspond to expressed phenotypes (41). Regardless of specific outcome, these results clearly demonstrate that the APE assay allows for cross-domain comparisons of growth on a variety of environmentally relevant substrates.

Overall, the novel APE assay presents several advantages over the current polysaccharide degradation assays. This method is less time-consuming and more cost-effective, as it can be prepared 24 hours before use with a significantly lower concentration of substrate. The APE assay also simplifies microplate preparation due to easier substrate loading and an AP that enables quick and precise extraction of agar plugs. Cell growth and enzymatic activity assays have demonstrated that the APE assay is compatible with a wide range of complex insoluble substrates of fine particle size and has utility both across and within different domains and media systems, illustrating its potential to be expanded to further *in vivo* studies. Furthermore, due to a larger dynamic range and lower variability, we have demonstrated the utility of this high-throughput assay for identifying mutant phenotypes, expanding our current understanding of CAZyme activity during complex polysaccharide degradation. The APE is not without limitations, with one being the requirement for soluble (secreted) enzymes to be produced by a microbe. Bacteria and yeasts that require attachment to an insoluble substrate for degradation will not be amenable to using APE assay methods. Additionally, marine bacteria that produce agarases would also not be suitable for APE assays due to their ability to dissolve the solidifying matrix. Despite these caveats, the APE assay provides an effective and straightforward approach to studying microbial insoluble polysaccharide degradation in a high-throughput manner.

## SUPPLEMENTAL MATERIAL

File S1 spectrum.04171-25-s0001.stl Stereolithography file for 3D printing.

Supplemental tables spectrum.04171-25-s0002.pdf Tables S1 to S4.

ASM does not own the copyrights to Supplemental Material that may be linked to, or accessed through, an article. The authors have granted ASM a non-exclusive, world-wide license to publish the Supplemental Material files. Please contact the corresponding author directly for reuse.
