# Supplementary material for: Development of a high-throughput growth assay for bacteria or yeasts using an agar-based insoluble carbon source immobilization method
Source: Microbiol Spectr. 2026 Apr 30;14(6):e04171-25. doi: 10.1128/spectrum.04171-25 (PMC13227985; doi:10.1128/spectrum.04171-25)
Supplement: Supplemental tables — Tables S1 to S4. [file spectrum.04171-25-s0002.pdf]

Development of a high-throughput growth assay for bacteria or yeasts using an agar-based insoluble carbon source immobilization method

Jiabao Liang*[1], Isabella S. Naimi*[1], Achala Narayanan[2], François Maillard[3], Peter G. Kennedy[2], and Jeffrey G. Gardner[#1]

*These authors contributed equally to the manuscript. Co-lead author order was determined alphabetically by last name.

Running Title
3D printed agar pipette for insoluble substrate screening

Keywords
3D Printing, Carbohydrate, *Cellvibrio japonicus*, Chitin, Glycan, Pectin, Polysaccharide

Author Affiliations
[1]Department of Biological Sciences, University of Maryland - Baltimore County, Baltimore, Maryland, USA
[2]Department of Plant and Microbial Biology, University of Minnesota, St. Paul, Minnesota, USA
[3]Department of Biology, Lund University, Lund, Sweden

[#]Correspondence
Jeffrey G. Gardner
Department of Biological Sciences
University of Maryland - Baltimore County
Email: jgardner@umbc.edu
Phone: 410-455-3613
Fax: 410-455-3875

Table S1A. Laboratory strains and plasmids used in this study.

Strain Name	Genotype	Source / Reference
C. japonicus Ueda107	Prototroph (wild-type)	Laboratory collection
C. japonicus Δ*gsp* (CJA_3325-3333)	*C. japonicus Udea107* Δ*gsp*	(1)
C. japonicus Δ*bgl2A* (CJA_0496)	*C. japonicus Udea107* Δ*bgl2A*	This study
C. japonicus Δ*cbp2D* (CJA_2616)	*C. japonicus Udea107* Δ*cbp2D*	(2)
C. japonicus Δ*cbp2E* (CJA_2615)	*C. japonicus Udea107* Δ*cbp2E*	(2)
C. japonicus Δ*cbp2D* Δ*cbp2E*	*C. japonicus Udea107* Δ*cbp2D* Δ*cbp2E*	This study
E. coli DH5α	λ^- ϕ80d*lacZ*ΔM15 Δ(*lacZYA-argF*) *U169 recA1 endA1 hsdR17*(r_K^- m_K^-) *supE44 thi-1 gyrA relA1*	Laboratory collection
E. coli S17 λpir	Tp^r Sm^r *recA thi pro hsdR hsdM*$^+$ RP4-2-Tc::Mu::Km Tn*7* λ*pir*	Laboratory collection
pK18*mobsacB*	pMB1, *mob*$^+$, *sacB*$^+$, Km^r	(3)
pRK2013	ColE1, RK2-Mob^+, RK2-Tra^+, Km^r	(4)

Table S1B: Environmental isolates used in this study.

Strain Name	Classification	Source / Reference
Delftia acidovorans sp. 105	Bacteria	Laboratory collection
Pseudomonas rhodesiae sp. 90	Bacteria	Laboratory collection
Pedobacter sp. 107	Bacteria	Laboratory collection
Flavobacteria sp. 150	Bacteria	Laboratory collection
Vanrija sp. AA21	Yeast	Laboratory collection
Candida railenensis sp. AA4	Yeast	Laboratory collection
Cryptococcus sp. 116	Yeast	Laboratory collection
Apiotrichum porosum sp. 34	Yeast	Laboratory collection

Table S2. Growth dynamics of *C. japonicus* wild-type and Δ*gsp* mutant strains on MOPs defined media supplemented with 0.2% (w:v) glucose, 0.2% (w:v) barley β-glucans, 0.2% (w:v) potato starch, or 2% (w:v) autoclaved yeast cells. Data shown here are from the experiment shown in **Figure 3**.

**Substrate & Method**	**Strain**	**Time Points (T_i and T_f)**	**Growth Rate (gen/hr)**	**Lag Time (hours)**	**Average Max OD_{600}**
Glucose (AP)	*C. japonicus WT*	3, 9	0.21 ± 0.03	2.5	1.26 ± 0.07
	C. japonicus Δ*gsp*	3, 10	0.19 ± 0.03	2.25	1.12 ± 0.08
Glucose (ACS)	*C. japonicus WT*	6, 11	0.28 ± 0.05	4.25	0.72 ± 0.05
	C. japonicus Δ*gsp*	8, 14	0.2 ± 0.01	6.25	0.73 ± 0.24
Barley β-glucans (AP)	*C. japonicus WT*	5, 9	0.32 ± 0.05	4	1.11 ± 0.15
	C. japonicus Δ*gsp*	12, 15	0.14 ± 0.07	9.75	0.38 ± 0.13
Barley β-glucans (AP)	*C. japonicus WT*	7, 12	0.23 ± 0.06	5.5	0.61 ± 0.04
	C. japonicus Δ*gsp*	13, 17	0.11 ± 0.04	12.75	0.35 ± 0.05
Potato Starch (AP)	*C. japonicus WT*	6, 9	0.4 ± 0.04	5.25	1.02 ± 0.08
	C. japonicus Δ*gsp*	N/A	N/A	N/A	0.14 ± 0.02
Potato Starch (ACS)	*C. japonicus WT*	7, 11	0.29 ± 0.05	6.75	0.67 ± 0.04
	C. japonicus Δ*gsp*	N/A	N/A	N/A	0.12 ± 0.02
Yeast Cells (AP)	*C. japonicus WT*	5, 9	0.21 ± 0.04	N/A	1 ± 0.07
	C. japonicus Δ*gsp*	N/A	N/A	23.5	0.28 ± 0.03
Yeast Cells (ACS)	*C. japonicus WT*	10, 14	0.09 ± 0.02	8.75	0.31 ± 0.05
	C. japonicus Δ*gsp*	N/A	N/A	N/A	0.12 ± 0.01

Table S3. Growth dynamics of *C. japonicus* wild-type, Δ*gsp,* and Δ*bgl2A* mutant strains on MOPs defined media supplemented with 0.2% (w:v) glucose, 0.2% (w:v) pectin from apple, or 0.2% (w:v) galactan from potato. Data shown here are from the experiment shown in **Figure 4**.

**Substrate**	**Strain**	**Time Points (T_i and T_f)**	**Growth Rate (gen/hr)**	**Lag Time (hours)**	**Average Max OD_{600}**
Glucose	*C. japonicus WT*	5, 10	0.35 ± 0.05	4	1.21 ± 0.05
	C. japoncius Δgsp	8, 14	0.17 ± 0.04	3.25	0.83 ± 0.16
	C. japonicus Δbgl2A	5, 10	0.35 ± 0.05	4.25	1.19 ± 0.05
Pectin (Apple)	*C. japonicus WT*	6, 10	0.27 ± 0.01	5.5	0.73 ± 0.09
	C. japoncius Δgsp	5, 7	0.07 ± 0.03	5	0.18 ± 0.02
	C. japonicus Δbgl2A	6, 10	0.3 ± 0.02	5.5	0.8 ± 0.11
Galactan (Potato)	*C. japonicus WT*	5, 9	0.37 ± 0.03	5	0.83 ± 0.03
	C. japoncius Δgsp	7, 13	0.23 ± 0.02	4.5	0.83 ± 0.03
	C. japonicus Δbgl2A	13, 16	0.26 ± 0.03	5.25	0.8 ± 0.04

Table S4. Growth dynamics of *C. japonicus* wild-type, Δ*gsp,* and Δ*cbp2D/E* single and double mutant strains on MOPs defined media supplemented with 0.2% (w:v) glucose, 1% (w:v) filter paper, 1% (w:v) beta-chitin from squid pen, 0.2% (w:v) xylan, 0.2% (w:v) pectin from apple, or 0.2% (w:v) autoclaved yeast cells using either test tube with BCD or microplate with AP assay. Data shown here are from the experiment shown in **Figure 5**.

**Substrate**	**Strain**	**Time Points (T_i & T_f)**	**Growth Rate (gen/hr)**	**Lag Time (hours)**	**Average Max OD_{600}**
Glucose	*C. japonicus WT*	3, 7	0.46 ± 0.02	3	1.48 ± 0.02
	C. japoncius Δgsp	4, 7	0.35 ± 0.04	3	1.02 ± 0.03
	C. japonicus Δcbp2D	3, 7	0.43 ± 0.03	3	1.48 ± 0.02
	C. japonicus Δcbp2E	3, 7	0.42 ± 0.01	2	1.54 ± 0.08

	C. japonicus ΔCbp2DΔcbp2E	3, 7	0.43 ± 0.02	3	1.49 ± 0.02
Filter Paper	*C. japonicus WT*	24, 53	0.06 ± 0.01	24	0.6 ± 0.03
	C. japoncius Δgsp	N/A	N/A	N/A	0.01 ± 0.01
	C. japonicus Δcbp2D	53, 77	0.03 ± 0.01	53	0.34 ± 0.22
	C. japonicus Δcbp2E	53, 77	0.03 ± 0.01	48	0.27 ± 0.18
	C. japonicus Δcbp2DΔcbp2E	53, 77	0.04 ± 0.01	53	0.24 ± 0.2
Beta-Chitin	*C. japonicus WT*	5, 10	0.32 ± 0.04	4.75	0.91 ± 0.09
	C. japoncius Δgsp	8, 13	0.14 ± 0.02	5.25	0.66 ± 0.07
	C. japonicus Δcbp2D	5, 9	0.33 ± 0.04	4.5	0.98 ± 0.11
	C. japonicus Δcbp2E	5, 9	0.33 ± 0.05	4.25	0.95 ± 0.07
	C. japonicus Δcbp2DΔcbp2E	5, 9	0.28 ± 0.03	4.25	0.99 ± 0.05
Xylan	*C. japonicus WT*	6, 10	0.2 ± 0.06	5.5	0.98 ± 0.08
	C. japoncius Δgsp	N/A	N/A	7.25	0.27 ± 0.06
	C. japonicus Δcbp2D	6, 10	0.19 ± 0.05	5.5	0.96 ± 0.08
	C. japonicus Δcbp2E	6, 9	0.17 ± 0.02	5	0.96 ± 0.08
Pectin from Apple	*C. japonicus Δcbp2DΔcbp2E*	6, 10	0.19 ± 0.05	5.25	0.91 ± 0.08
	C. japonicus WT	8, 12	0.1 ± 0.04	7.25	1.01 ± 0.08
	C. japoncius Δgsp	N/A	N/A	14.25	0.3 ± 0.09
	C. japonicus Δcbp2D	8, 12	0.17 ± 0.03	7.5	1.1 ± 0.08
	C. japonicus Δcbp2E	7, 13	0.1 ± 0.03	7	1.17 ± 0.07
Yeast Cells	*C. japonicus Δcbp2DΔcbp2E*	8, 13	0.16 ± 0.07	7	1.15 ± 0.11
	C. japonicus WT	5, 10	0.32 ± 0.04	4.75	0.91 ± 0.09

	C. japoncius Δgsp	8, 13	0.14 ± 0.02	5.25	0.66 ± 0.07
	C. japonicus Δcbp2D	5, 9	0.33 ± 0.04	4.5	0.98 ± 0.11
	C. japonicus Δcbp2E	5, 9	0.33 ± 0.05	4.25	0.95 ± 0.07
	C. japonicus Δcbp2DΔcbp2E	5, 9	0.28 ± 0.03	4.25	0.99 ± 0.05

Additional Supplemental Files

Supplemental File 1 – Stereolithography file for 3D printing

REFERENCES

1. Nelson CE, Gardner JG. 2015. In-Frame Deletions Allow Functional Characterization of Complex Cellulose Degradation Phenotypes in *Cellvibrio japonicus*. *Applied and Environmental Microbiology*. 81(17):5968-75.
2. Gardner J, Crouch L, Labourel A, Forsberg Z, Bukhman Y, Vaaje-Kolstad G, Gilbert H, Keating D. 2014. Systems biology defines the biological significance of redox-active proteins during cellulose degradation in an aerobic bacterium. *Molecular Microbiology*. 95(5):1121-33.
3. Schafer A, Tauch A, Jager W, Kalinowski J, Thierbach G, Puhler A. 1994. Small mobilizable multi-purpose cloning vectors derived from the *Escherichia coli* plasmids pK18 and pK19: selection of defined deletions in the chromosome of *Corynebacterium glutamicum. Gene.* 145(1):69-73.
4. Figurski DH, Helinsk DR. 1979. Replication of an origin-containing derivative of plasmid RK2 dependent on a plasmid function provided in trans. *Proceedings of the National Academy of Sciences of the United States of America*. 76(4):1648-52.
